# Tumor size and proliferative marker geminin rather than Ki67 expression levels significantly associated with maximum uptake of ^18^F-deoxyglucose levels on positron emission tomography for breast cancers

**DOI:** 10.1371/journal.pone.0184508

**Published:** 2017-09-08

**Authors:** Arisa Nishimukai, Natsuko Inoue, Ayako Kira, Masashi Takeda, Koji Morimoto, Kazuhiro Araki, Kazuhiro Kitajima, Takahiro Watanabe, Seiichi Hirota, Toyomasa Katagiri, Shoji Nakamori, Kouhei Akazawa, Yasuo Miyoshi

**Affiliations:** 1 Department of Surgery, Division of Breast and Endocrine, Hyogo College of Medicine, Nishinomiya City, Hyogo, Japan; 2 Department of Pathology, Yao Municipal Hospital, Yao City, Osaka, Japan; 3 Department of the Science of Living, Osaka Women’s Junior College, Fujiidera City, Osaka, Japan; 4 Department of Surgery, National Hospital Organization Osaka National Hospital, Chuo-ku, Osaka, Japan; 5 Department of Nuclear Medicine and PET Center, Hyogo College of Medicine, Nishinomiya City, Hyogo, Japan; 6 Department of Surgical Pathology, Hyogo College of Medicine, Nishinomiya City, Hyogo, Japan; 7 Division of Genome Medicine, Institute for Genome Research, Tokushima University, Kuramoto-cho, Tokushima, Japan; 8 Department of Medical Informatics, Niigata University Medical & Dental Hospital, Chuo-ku, Niigata, Japan; Seconda Universita degli Studi di Napoli, ITALY

## Abstract

It has been well established that maximum standardized uptake value (SUVmax) for ^18^F-fluorodeoxyglucose positron-emission tomography/computed tomography (FDG PET/CT) is clinically useful for evaluating treatment efficacy as well as predicting prognosis of breast cancer patients. Although SUVmax reflects increased glucose uptake and metabolism possibly induced by activation of growth factor signaling or TP53 dysfunction, tumor characteristics of SUVmax-high breast cancers remain to be elucidated. For the present study, we used immunohistochemical staining to investigate expressions of phospho-ribosomal protein S6 (pS6, downstream molecule of phosphatidyl inositol 3-kinase/Akt/mammalian target of the rapamycin/S6K pathway) and phosphor-p44/42 mitogen-activated protein kinase (pMAPK). Expression levels of TP53 and proliferative marker geminin as well as Ki67 were also examined by means of immunostaining in 163 invasive breast cancers. Cutoff values were set at 10% for pS6, 20% for pMAPK and TP53, and 4% for geminin. The SUVmax levels were significantly higher in the pS6-positive (p = 0.0173), TP53-positive (p = 0.0207) and geminin-high cancers (p<0.0001), but there was no significant association between pMAPK expression levels and SUVmax levels. Multivariable analysis showed that a high geminin level (odds ratio: 6.497, 95% confidence interval: 2.427–19.202, p = 0.0001) and large tumor size (6.438, 2.224–20.946, p = 0.0005) were significantly and independently associated with SUVmax-high. Univariable but not multivariable analysis indicated that Ki67-high significantly correlated with SUVmax-high. Twenty of 23 (87.0%) breast cancers with tumor size >2cm and geminin-high showed SUVmax-high, while only 6 of 49 (12.2%) breast cancers ≤2cm in size and with low geminin levels were SUVmax-high. In conclusion, we could determine that breast cancers with a large tumor and a geminin-high rather than Ki67-high proliferative marker were significantly associated with high levels of SUVmax. These findings may signify that SUVmax reflects tumor characteristics with high proliferative activity but not activation of mTOR/S6K and MAPK pathways or increased glucose metabolism due to dysfunction of TP53.

## Introduction

^18^F-fluorodeoxyglucose positron-emission tomography/computed tomography (FDG PET/CT) has been widely used in daily clinical practice as an imaging tool for detecting primary as well as metastatic breast cancers [1, 2]. In addition to this diagnostic modality, many studies have demonstrated that the maximum standardized uptake value (SUVmax) on FDG PET has predictive value for prognosis of operable breast cancers [3–7]. In addition, the value of SUVmax as a predictive tool for treatment efficacy has been demonstrated in several studies which found that early reduction of SUVmax resulted in improvement leading to pathological complete response for breast cancers treated with neoadjuvant chemotherapy [8, 9]. Similarly, early responses determined in terms of SUVmax are reportedly associated with good prognosis for breast cancer patients treated with neoadjuvant chemotherapy [10, 11].

While the details of the mechanisms of prognostic or predictive values remain to be elucidated, it is speculated that the mechanism of the link between SUVmax and prognosis or treatment efficacy might be related to differences in glucose metabolism in cancer cells [12]. It is also conceivable that SUVmax reflects glucose uptake in cancer cells and is possibly regulated by functioning of the glucose transporter (GLUT) present in membrane. Since expression of GLUT-1 and glucose metabolism are likely to be regulated by activation of growth factor signaling, including the phosphatidyl inositol 3-kinase (PI3K)/Akt/mammalian target of the rapamycin (mTOR) pathway [13, 14] and the mitogen-activated protein kinase (MAPK) pathway [15], the baseline value of or changes in SUVmax after treatment may reflect activation or suppression of such growth factor signaling. In addition, TP53 has been demonstrated to have a role in controlling glucose metabolism by downregulation of GLUT expression through the inhibition of nuclear factor kappa-B kinase [16]. Since TP53 function is frequently lost due to its mutation occurring in breast cancers [17], it may be speculated that upregulation of SUVmax levels is related to dysfunction of TP53. Furthermore, it has been reported that higher uptake of FDG is significantly associated with proliferative ability including increases in mitotic counts or the Ki67 labeling index [7, 18–20]. Although the existence of connections between SUVmax levels and activation of growth factor signaling, dysfunction of TP53 or proliferative activity are speculative, hardly any studies concerning this issue in breast cancers have been reported. Thus, identification and clarification of these characteristics in breast cancers with high levels of SUVmax remain a quite important issue in clinical practice.

The aim of the present study was thus to identify factors associated with SUVmax levels focusing on growth factor signaling, glucose metabolism and proliferative activity. To determine PI3K/Akt/mTOR and MAPK pathways activation, immunohistochemical staining was used to evaluate phospho-ribosomal protein S6 (pS6, a downstream molecule of the PI3K/Akt/mTOR/S6K pathway and phosphorylated by S6K), and phosphor-p44/42 MAPK (pMAPK) and TP53 for detecting alterations in TP53 proteins as an indicator of glucose metabolism. In addition, proliferative marker geminin, which is expressed selectively during S to M phases in the cell cycle [21], as well as Ki67 was investigated immunohistochemically in order to assess correlations between proliferative activity and SUVmax levels.

## Materials and methods

### Recruitment of patients and pathological diagnosis

For this retrospective study, 700 breast cancer patients who underwent surgery at the Hyogo College of Medicine Hospital between May 2008 and May 2014 were consecutively recruited. Of these 700 patients, 565 had invasive cancers, for 409 of which preoperative data for ^18^F-FDG PET/CT imaging were available, and from 377 of the latter written informed consent was obtained for participation in this study. For 89 of the 377 cases clinical data were lacking, including for estrogen receptor (ER), progesterone receptor (PgR), human epidermal growth factor receptor 2 (HER2), nuclear grade or tumor size. Moreover, tumor tissues available were insufficient due to small tumor size obtained during operation (n = 62) or core needle biopsy before the start of preoperative therapy (n = 63). Of the remaining 163 samples were selected for the present study. Samples obtained by core needle biopsy prior to therapy were used for patients treated with preoperative chemotherapy (n = 31) and endocrine therapy (n = 18) and intra-operatively resected tissues for the remaining cases. Nuclear grading was done in accordance with the criteria defined by the Japanese Breast Cancer Society classification [22]. Classification as positive for ER and PgR was based on nuclear staining of these receptors in 1% or more of the tumor cells. For membrane staining of HER2, a score of 3 was judged to indicate HER2 positivity, while a score of 2 with positivity for fluorescence in situ hybridization (FISH) was also classified as HER2-positive.

Details of the staining procedure and antibodies used for staining of ER, PgR, HER2 and Ki67 were described previously [23]. The Ethics Committee of Hyogo College of Medicine approved the present study (No. 106) and written informed consent was obtained from all 163 participants.

### Immunohistochemical staining procedure

Formalin-fixed, paraffin-embedded tumor tissues obtained intraoperatively or by core needle biopsy prior to treatment were used for further immunohistochemical staining. To avoid protein degradation, the tissues were fixed in buffered formalin immediately after resection and fixed for 24 to 48 hours. For pS6, pMAPK and TP53 staining, we used primary antibodies D57.2.2E (phospho-S6 ribosomal protein [Ser235/236], rabbit monoclonal antibody; Cell Signaling Technology, Danvers, MA), D13.14.4E (phospho-p44/42 MAPK [Thr202/Tyr204], rabbit monoclonal antibody; Cell Signaling Technology) and DO-7 (mouse monoclonal antibody; Dako, Glostrup, Denmark), respectively. The primary antibodies were diluted 1:200 for pS6 and pMAPK, and 1:100 for TP53. Details of the methods used for immunostaining of pS6, pMAPK and TP53 have been described in a previous report [23]. For immunohistochemical staining of geminin, a rabbit anti-Geminin antibody (FL-209, diluted 1:200; Santa Cruz Biotechnology, Santa Cruz, CA) was used by following a method described elsewhere [24].

Moderate to intense staining in cytoplasm for pS6 and in cytoplasm and nuclei for pMAPK were evaluated as described in a previous study [23]. Strong nuclear staining was assessed for TP53 and geminin expression [23, 24]. We counted 500 cancer cells selected in different areas of the stained lesions. The slides were examined by three of the authors (A.N., A.K. and Y.M.) who were unaware of the clinical and FDG PET data and in case of discrepancy the samples were further evaluated in order to reach a consensus.

### ^18^F-FDG PET/CT imaging and determination of SUVmax

Whole-body ^18^F-FDG PET examinations with a CT scanner (Gemini GXL16; Philips Medical Systems, Eindhoven, The Netherlands) were performed at Hyogo College of Medicine Hospital. As described in a previous study [25], 4.0 MBq/kg body weight of ^18^F-FDG was used for PET and the scanning image was obtained approximately 60 min after the injection. The SUV was calculated as the regional radioactivity concentration (Bq/mL)/[injected dose (Bq)/patient’s weight (g)] in the most intense area of ^18^F-FDG accumulation (a region of interest: ROI) and the peak SUV in the pixel with the highest count within the ROI was defined as the SUVmax. The cutoff value for SUVmax-high and -low was set at 3.585, which was determined in our previous study to identify relapse-free survival in 387 breast cancer patients, including the cases in the current study [7].

### Statistical analysis

Associations between SUVmax levels and clinicopathological characteristics or immunohistochemically determined factors were analyzed with the chi-square test or Fisher’s exact test as appropriate. Comparison between SUVmax levels and pS6, pMAPK, TP53 or geminin labeling indices were analyzed with the Spearman correlation coefficient, while SUVmax levels for different groups were compared with the Mann-Whitney test or Kruskal-Wallis test. We used univariable and multivariable logistic regression analyses to determine the associations between SUVmax and clinical or immunohistochemically determined factors. The variables were included in the multivariable model when statistical significance was obtained for a stepwise forward selection. Odds ratios (ORs) and the corresponding 95% confidence intervals (CIs) were also calculated. Differences were considered statistically significant for p<0.05. JMP Pro 11 software (SAS Institute Inc., Cary, NC) was used for all statistical analyses.

## Results

### Immunohistochemical staining of pS6, pMAPK, TP53, geminin and Ki67 expression levels and correlations of these factors with SUVmax levels

**Fig 1:**
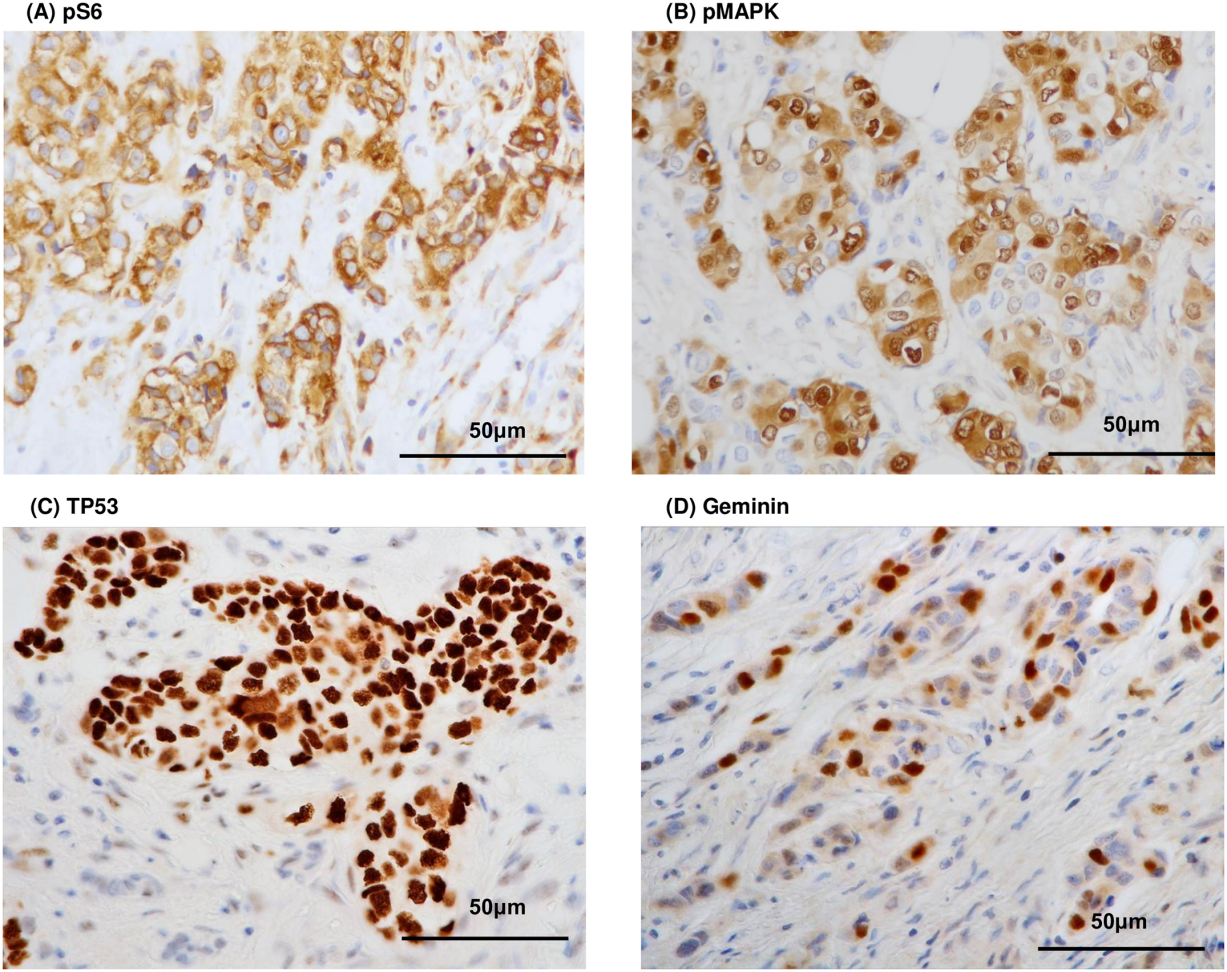
Representative positive immunostaining for pS6 (A), pMAPK (B), TP53 (C) and geminin (D). Staining of cytoplasm (pS6), cytoplasm and nuclei (pMAPK) and nuclei (TP53 and geminin) was evaluated.

**Fig 2:**
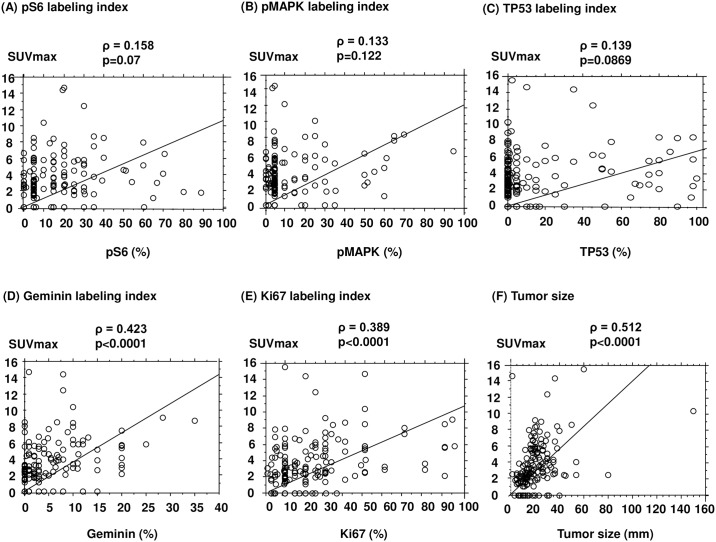
Correlations between SUVmax levels and pS6 (A), pMAPK (B), TP53 (C), geminin (D) or Ki67 (E) labeling indices and tumor size (F).

Activation of PI3K/Akt/mTOR and MAPK pathways was evaluated in terms of pS6 and pMAPK expression levels determined by immunohistochemical staining as represented in Fig 1(A) and 1(B). Representative positive staining of TP53 and geminin is also shown in Fig 1(C) and 1(D). The expression levels of geminin and Ki67 were significantly associated with SUVmax levels (correlation coefficient (ρ): 0.423, p<0.0001; Fig 2D for geminin and ρ: 0.389, p<0.0001; Fig 2E for Ki67). However, no significant associations between SUVmax levels and pS6 (p = 0.07), pMAPK (p = 0.122) or TP53 (p = 0.0869) were found as shown in Fig 2(A)–2(C). The significant and positive relationship between tumor size and SUVmax levels is shown in Fig 2(F) (ρ: 0.512, p<0.0001).

### Determination of optimal cutoff values of pS6, pMAPK, TP53, geminin or Ki67 for SUVmax levels

**Fig 3:**
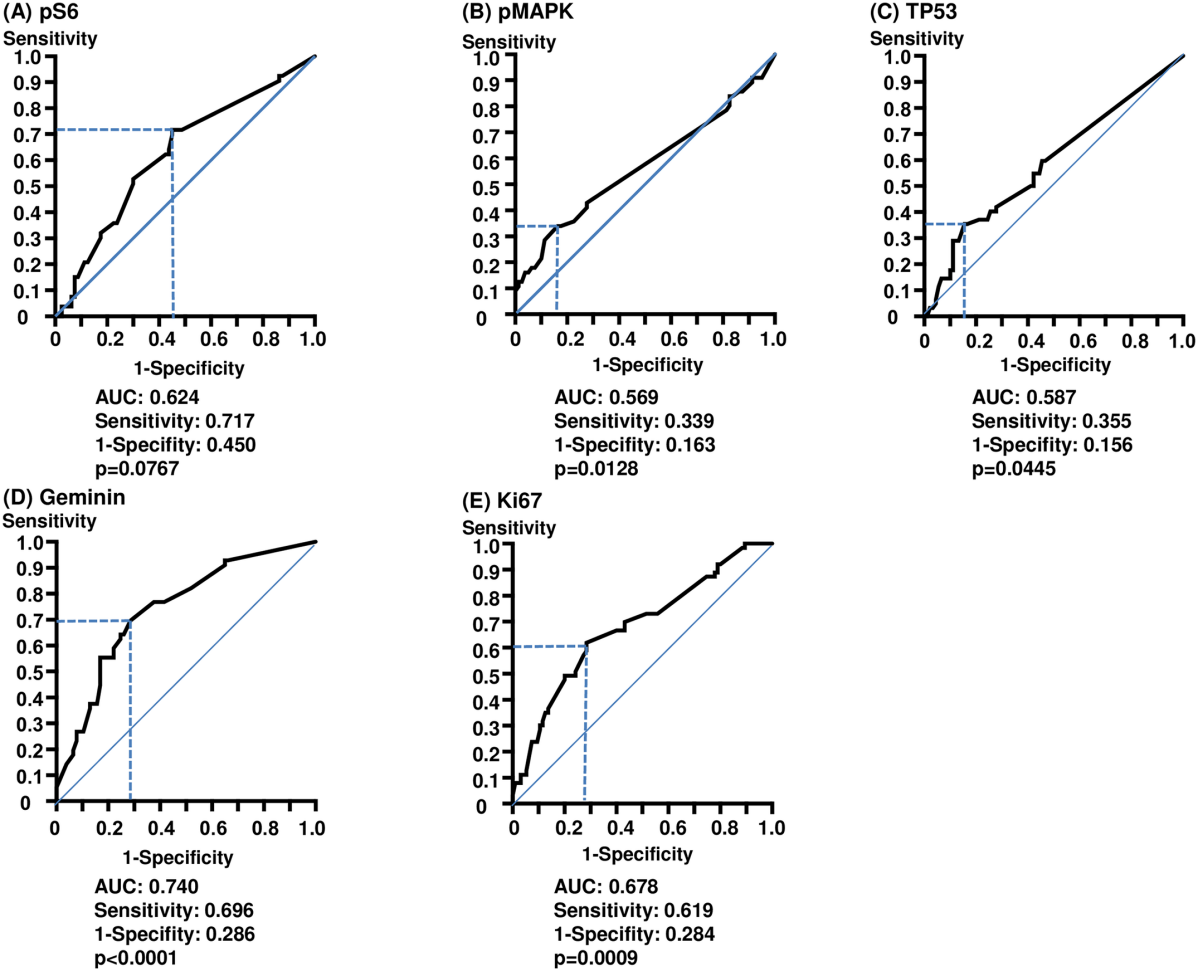
Receiver operating characteristics curves of pS6, pMAPK, TP53, geminin and Ki67 for SUVmax. The cutoff values were determined as 10% for pS6 (A), 20% for pMAPK (B), 20% for TP53 (C), 4% for geminin (D) and 21.5% for Ki67 (E).

In order to identify optimal cutoff values for immunohistochemical markers, we used receiver operating characteristics curves calculated with the Youden index for the areas under the curve (AUC). As shown in Fig 3(A)–3(E), cutoffs values were determined as 10% (AUC: 0.624, p = 0.0767) for pS6, 20% (AUC: 0.569, p = 0.0128) for pMAPK, 20% (AUC: 0.587, p = 0.0445) for TP53, 4% (AUC: 0.740, p<0.0001) for geminin and 21.5% (AUC: 0.678, p = 0.0009) for Ki67. Sensitivity and 1-specificity for geminin were 0.696 and 0.286, respectively. These cutoff values were used for subsequent analyses.

### Correlations between SUVmax levels and clinicopathological or immunohistochemical factors

Since the optimal cutoff value of SUVmax for relapse-free survival of operated breast cancer patients was determined at 3.585 in a previous study of ours [7], we used this cutoff value to divide SUVmax levels into two groups (SUVmax-high: n = 66, SUVmax-low: n = 97). As shown in Table 1, SUVmax-high cancers were significantly more frequent in cancers with a large tumor size (>2cm, p<0.0001), nuclear grade 3 (p<0.0001), ER-negative (p = 0.0010), PgR-negative (p = 0.0013) and high Ki67 expression levels (p<0.0001). SUVmax-high cancers were detected significantly higher more frequently in pS6-positive, TP53-positive and geminin-high breast cancers (p = 0.0173, p = 0.0207 and p<0.0001, respectively). On the other hand, there was only a marginally significant association between SUVmax and pMAPK positivity (p = 0.0568).

**Table 1:** Relationships between SUVmax levels and clinicopathological or immunohistochemical factors of breast cancers.

Characteristics	SUVmax-high^a^ (n = 66)	SUVmax-low^a^ (n = 97)	p-value
Menopausal status			
Premenopausal	24 (43.6%)	31 (56.4%)	0.559
Postmenopausal	42 (38.9%)	66 (61.1%)	
Histological type			
No special	64 (41.8%)	89 (58.2%)	0.204
Special	2 (20.0%)	8 (80.0%)	
Tumor size			
≤2cm	29 (28.2%)	74 (71.8%)	<0.0001
>2cm	37 (61.7%)	23 (38.3%)	
Lymph node metastasis			
Negative	38 (35.5%)	69 (64.5%)	0.1128
Positive	27 (51.9%)	25 (48.1%)	
Not evaluated^b^	1 (25.0%)	3 (75.0%)	
Nuclear grade			
1+2	43 (32.3%)	90 (67.7%)	<0.0001
3	23 (76.7%)	7 (23.3%)	
Estrogen receptor			
Positive	47 (34.6%)	89 (65.4%)	0.0010
Negative	19 (70.4%)	8 (29.6%)	
Progesterone receptor			
Positive	36 (32.1%)	76 (67.9%)	0.0013
Negative	30 (58.8%)	21 (41.2%)	
HER2 status			
Negative	53 (37.6%)	88 (62.4%)	0.0648
Positive	13 (59.1%)	9 (40.9%)	
Ki67 expression levels^c^			
Low	24 (26.1%)	68 (73.9%)	<0.0001
High	39 (59.1%)	27 (40.9%)	
Unknown	3 (60.0%)	2 (40.0%)	
pS6 expression^d^			
Negative	16 (26.7%)	44 (73.3%)	0.0173
Positive	37 (50.7%)	36 (49.3%)	
Unknown	13 (43.3%)	17 (56.7%)	
pMAPK expression^e^			
Negative	37 (35.6%)	67 (64.4%)	0.0568
Positive	19 (59.4%)	13 (40.6%)	
Unknown	10 (37.0%)	17 (63.0%)	
TP53 expression^e^			
Negative	40 (34.5%)	76 (65.5%)	0.0207
Positive	22 (61.1%)	14 (38.9%)	
Unknown	4 (36.4%)	7 (63.6%)	
Geminin expression levels^f^			
Low	17 (23.6%)	55 (76.4%)	<0.0001
High	39 (63.9%)	22 (36.1%)	
Unknown	10 (33.3%)	20 (66.7%)	

^a^ SUVmax (maximum standardized uptake value) high: ≥3.585, low: <3.585.

^b^ axillary examination was not performed.

^c^ low: <21.5%, high: ≥21.5%.

^d^ negative: <10%, positive: ≥10%.

^e^ negative: <20%, positive: ≥20%.

^f^ low: <4%, high: ≥4%.

### Univariable and multivariable analyses for SUVmax

Univariable analysis demonstrated that large tumor size (p<0.0001), lymph node metastasis-positivity (p = 0.0492), nuclear grade 3 (p<0.0001), ER-negativity (p = 0.0006), PgR-negativity (p = 0.0014), high levels of Ki67 (p<0.0001), pS6-positivity (p = 0.0045), pMAPK (p = 0.0174), TP53 (p = 0.0048) and high levels of geminin (p<0.0001) were significantly associated with high levels of SUVmax (Table 2). Variables which independently predict high levels of SUVmax were selected based on a stepwise forward regression model. Finally, a multivariable analysis determined that large tumor size (OR: 6.438, 95% CI: 2.224–20.946, p = 0.0005) and high levels of geminin (OR: 6.497, 95% CI: 2.427–19.202, p = 0.0001) were independent and significant predictive factors (Table 2).

**Table 2:** Univariable and multivariable analyses of clinical and immunohistochemical factors for SUVmax levels.

	n	Univariable analysis OR (95% CI)^a^	p-value	Multivariable analysis OR (95% CI)^a^	p-value
Menopausal status					
Premenopausal	55	1.00	0.56		
Postmenopausal	108	0.822 (0.425–1.595)			
T size					
≤2.0cm	103	1.00	<0.0001	1.00	0.0005
>2cm	60	4.105 (2.111–8.170)		6.438 (2.224–20.946)	
Lymph node metastasis					
Negative	107	1.00	0.0492		
Positive	52	1.961 (1.002–3.866)			
Nuclear grade					
1+2	133	1.00	<0.0001		
3	30	6.877 (2.863–18.486)			
Estrogen receptor status					
Positive	136	1.00	0.0006		
Negative	27	4.497 (1.889–11.627)			
Progesterone receptor status					
Positive	112	1.00	0.0014		
Negative	51	3.016 (1.532–6.046)			
HER2 status					
Negative	141	1.00	0.0585		
Positive	22	2.398 (0.969–6.178)			
Ki67 levels^b^					
Low	92	1.00	<0.0001		
High	66	4.093 (2.103–8.160)			
pS6^c^					
Negative	60	1.00	0.0045		
Positive	73	2.826 (1.375–5.997)			
pMAPK^d^					
Negative	104	1.00	0.0174		
Positive	32	2.647 (1.186–6.073)			
TP53^d^					
Negative	116	1.00	0.0048		
Positive	36	2.986 (1.394–6.583)			
Geminin levels^e^					
Low	72	1.00	<0.0001	1.00	0.0001
High	61	5.735 (2.744–12.467)		6.497 (2.427–19.202)	

^a^ Odds ratio (95% confidence interval).

^b^ low: <21.5%, high: ≥21.5%.

^c^ negative: <10%, positive: ≥10%.

^d^ negative: <20%, positive: ≥20%.

^e^ low: <4%, high: ≥4%.

### Comparison of SUVmax levels after combination of tumor size and geminin levels

**Fig 4:**
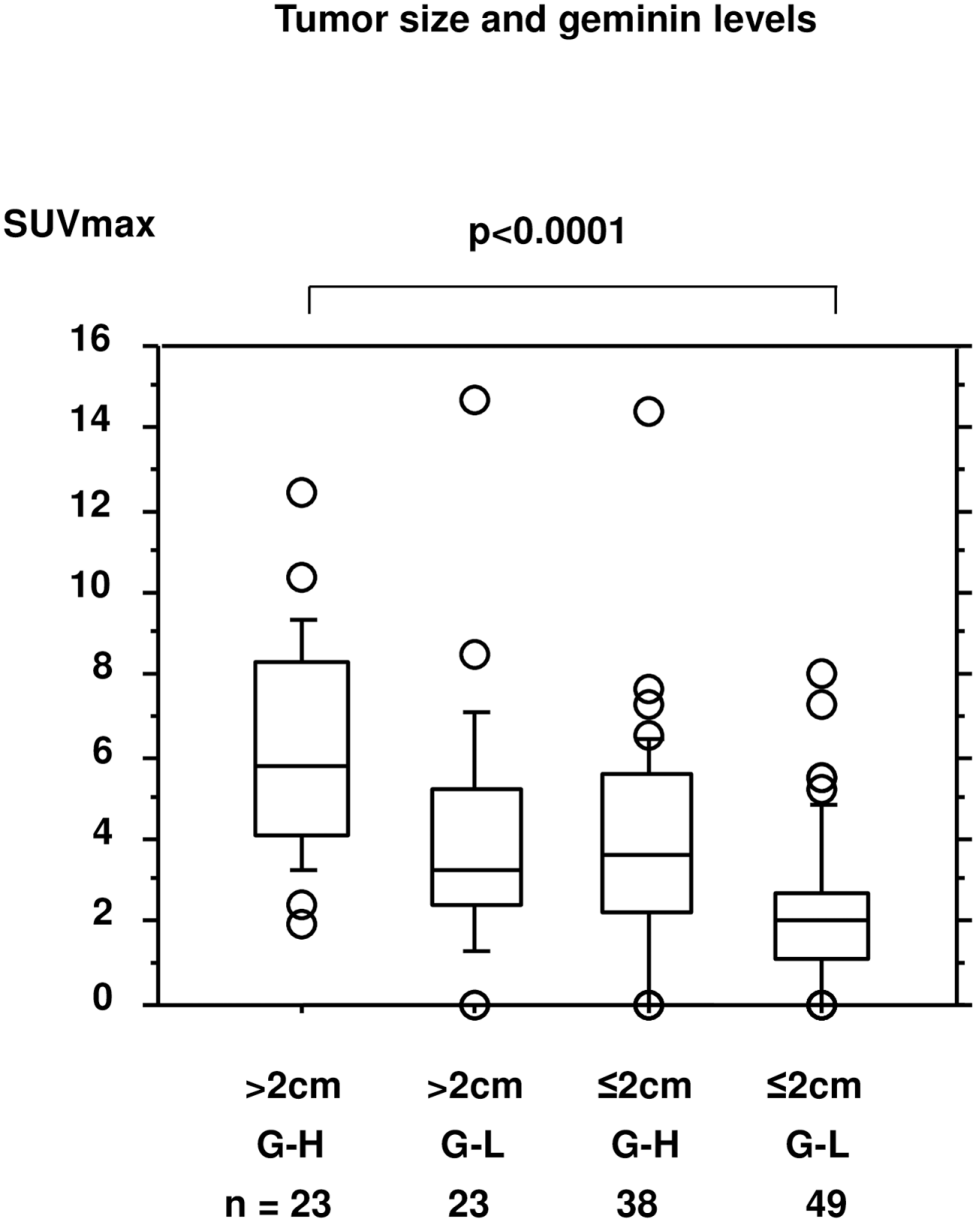
Comparison of SUVmax levels after combination of tumor size and geminin levels. SUVmax levels for groups with tumor size ≤2cm and geminin-low (G-L), ≤2cm and geminin-high (G-H), >2cm and geminin-low (G-L) and >2cm and geminin-high (G-H). Boxes represent median values and upper and lower quartiles.

Since tumor size and geminin expression levels were significant and independent in terms of likelihood of SUVmax-high levels, these factors were combined for further analysis of 133 breast cancers for which data on geminin were available. Since not enough breast cancer cells remained after other immunohistochemical examinations had been completed, evaluation of geminin in the remaining samples was not feasible. As expected, SUVmax levels were significantly different for the resultant four groups (p<0.0001, Fig 4). The SUVmax levels were highest for the tumor size (T) >2cm/geminin (Gem)-high group (median: 5.75, range: 1.91–12.4) and lowest for the T ≤2cm/Gem-low group (2.01, 0–8.0). SUVmax levels for the T >2cm/Gem-low (3.23, 0–14.68) and T ≤2cm/Gem-high (3.63, 0–14.42) showed intermediate levels. As shown in Table 3, 20 of the 23 T >2cm /Gem-high cancers (87.0%) were SUVmax-high and only 6 of the 49 T ≤2cm/Gem-low cancers (12.2%) were SUVmax-high when analyzed in all breast cancers (p<0.0001). Positive associations were consistently recognized in the ER-positive (p<0.0001) and HER2-negative (p<0.0001), and marginally in the HER2-positive (p = 0.0828) subsets.

**Table 3:** SUVmax levels according to tumor size and geminin expression levels.

Characteristics	SUVmax-high^a^ (n = 56)	SUVmax-low^a^ (n = 77)	p-value
All subsets			
T >2cm/Gem-high^b^	20 (87.0%)	3 (13.0%)	<0.0001
T >2cm/Gem-low	11 (47.8%)	12 (52.2%)	
T ≤2cm/Gem-high	19 (50.0%)	19 (50.0%)	
T ≤2cm/Gem-low	6 (12.2%)	43 (87.8%)	
ER-positive subset			
T >2cm/Gem-high	12 (85.7%)	2 (14.3%)	<0.0001
T >2cm/Gem-low	10 (47.6%)	11 (52.4%)	
T ≤2cm/Gem-high	16 (50.0%)	16 (50.0%)	
T ≤2cm/Gem-low	2 (4.8%)	40 (95.2%)	
ER-negative subset			
T >2cm/Gem-high	8 (88.9%)	1 (11.1%)	0.311
T >2cm/Gem-low	1 (50.0%)	1 (50.0%)	
T ≤2cm/Gem-high	3 (50.0%)	3 (50.0%)	
T ≤2cm/Gem-low	4 (57.1%)	3 (42.9%)	
HER2-negative subset			
T >2cm/Gem-high	13 (92.9%)	1 (7.1%)	<0.0001
T >2cm/Gem-low	8 (44.4%)	10 (55.6%)	
T ≤2cm/Gem-high	18 (48.6%)	19 (51.4%)	
T ≤2cm/Gem-low	5 (11.6%)	38 (88.4%)	
HER2-positive subset			
T >2cm/Gem-high	7 (77.8%)	2 (22.2%)	0.0828
T >2cm/Gem-low	3 (60.0%)	2 (40.0%)	
T ≤2cm/Gem-high	1 (100%)	0 (0%)	
T ≤2cm/Gem-low	1 (16.7%)	5 (83.3%)	

^a^ SUVmax (maximum standardized uptake value) high: ≥3.585, low: <3.585.

^b^ geminin (Gem) low: <4%, high: ≥4%.

## Discussion

The findings of this study have confirmed that expression levels of proliferative marker geminin and tumor size are significantly associated with SUVmax levels in operated breast cancers. On the other hand, results of the multivariable analysis showed no significant associations between SUVmax and pS6, pMAPK or TP53 positivity. Since both pS6 and pMAPK expression levels correlated with activation of, respectively, the PI3K/Akt/mTOR and MAPK pathways, it may be speculated that influences on SUVmax levels were not directly regulated solely by activation of these signaling pathways or metabolic changes possibly derived from TP53 dysfunction. On the basis of the findings of our study, we speculate that higher levels of SUVmax represent activation of glucose metabolism of breast cancers with large tumor size and high proliferative activity.

The clinical usefulness of SUVmax for predicting prognosis of early breast cancers has been well established [3–7]. In addition to its prognostic significance, recent discoveries for SUVmax have indicated its usefulness for predicting the efficacy of chemotherapies, especially early response during neoadjuvant chemotherapies [8, 9]. Although the details of the regulating mechanisms of SUVmax remain to be elucidated, SUVmax seems to reflect uptake of glucose into cancer cells and its metabolism. The SUVmax levels were significantly higher in our study’s subsets with a high nuclear grade, ER negativity, PgR negativity and high levels of Ki67. In spite of the unresolved issues, we were able to demonstrate that large tumor size and high proliferative activity determined by geminin expression were significant and independent predictive factors for high levels of SUVmax by multivariable analyses. Large and highly proliferative cancers may necessitate increased levels of glucose uptake and its metabolism may have resulted in higher levels of SUVmax. Consistent with our findings, Koo et al. reported that an increased uptake of ^18^F-FDG was significantly associated with a high Ki67 index and large tumor size in primary triple-negative breast cancers [26]. We were the first to demonstrate that proliferative marker geminin, but not Ki67, is closely linked with SUVmax. The relationship of SUVmax levels with proliferative activity and tumor size appears to be consistent in both the ER-positive and the HER2-negative subsets (Table 3). Although such a significant association could not be demonstrated in the ER-negative and the HER2-positive subsets, this outcome may not be conclusive due to the small number of samples (n = 24 and 21, respectively).

Interestingly, multivariable analysis showed geminin was superior to Ki67 for predicting SUVmax levels. Unlike Ki67, which is expressed from the G1 to M phase [27], geminin expression is detectable in the S, G2, and early M phase [21, 28]. We believe geminin is preferable to Ki67 evaluating the proliferative activity of breast cancer cells. Significant associations between SUVmax and expression of phosphorylated Akt and S6 have been identified in renal cell cancer [29], as have significant correlations between SUVmax and PI3K and pAkt in laryngeal [30] and cervical cancers [14]. In contrast to the discoveries of these studies, we could not find any significant association of SUVmax with either PI3K/Akt/mTOR or MAPK activation as determined by expression levels of pS6 or pMAPK, respectively. Similar to our result, one study reported no significant association between p53 status and increased uptake of ^18^F-FDG in primary triple-negative breast cancers [26]. Gao et al. reported that chemokine CCL5 increased cell surface expression of GLUT1 and that ATP production mediated through upregulated glucose metabolism resulted in enhanced proliferation of breast cancer cells [31]. We speculate, therefore, that enhanced SUVmax levels may be induced not by activation of growth factor signaling, but by other mechanisms including chemokines such as CCL5. This issue needs to be investigated in future studies of a larger number of samples including breast cancers.

We cannot exclude the possibility that in the small cancers SUVmax levels were not accurate as compared with actual uptake of FDG because of the partial volume effect [32]. However, since significant associations between geminin and SUVmax were recognized in cancers with both small and large tumors (Fig 4 and Table 3), we believe geminin is a useful marker for predicting SUVmax levels even when the partial volume effect is taken into consideration. However, the significance of geminin for FDG uptake needs to be investigated by using other 3D parameters such as metabolic tumor volume or total lesion glycolysis. Since SUVmax levels were measured during examination of clinical practice, we have no data concerning metabolic tumor volume or total lesion glycolysis. Considering that SUVmax is the most frequently used in daily practice, we believe that studies which analyzed relationships between SUVmax and immunohistochemical markers are still useful. Further investigation using these other 3D parameters needs to be done in future. Another limitation of the present study is the cutoff value for SUVmax for relapse-free survival of 3.585, which was determined in our previous study of 387 patients. Usually, cutoff values for SUVmax for predicting patients’ prognosis are set at 3, 4 or 5.6 [3, 4, 33]. However, it is difficult to directly apply cutoff values determined in other studies because such SUVmax values vary depending on the institute due to differences in PET/CT devices used, so that adjustment using phantom models is necessary when using cutoff values from other institutes. For this reason, we used a cutoff value of 3.585, which was determined on the basis of the 387 cases in our hospital, including the patients in the current study so that no adjustment was necessary. Nevertheless, we confirmed that, with different cutoff values of 3, 4 or 5.6, the significant and independent usefulness of tumor size and geminin was consistently recognized irrespective of cutoff values (data not shown). Since the results presented here were obtained in a retrospective, single-institute study and the sample size was not sufficiently large, they need to be validated in future prospective studies to deal with the issues mentioned above with a large number of patients.

In conclusion, we found that proliferative marker geminin and tumor size were significantly associated with SUVmax levels in operated breast cancers. Since our findings did not show any significant associations of SUVmax with pS6, pMAPK or TP53 status by the multivariable analysis, we speculate that activation of the PI3K/Akt/mTOR and MAPK pathways or increased glucose metabolism due to TP53 dysfunction may not be the only factors to influence SUVmax levels. The finding of the present study that SUVmax levels reflect proliferative activity of breast cancers may prove to be useful for a better understanding of the clinical significance of SUVmax as a prognostic as well as a predictive indicator.
